# Optimization of Infant Nutrition: Exploring Feeding Practices Among Indian Mothers

**DOI:** 10.7759/cureus.73142

**Published:** 2024-11-06

**Authors:** Dhanasekhar Kesavelu, Sudhan Dhanasekhar, Wasim Akram, Amanda Rachel, Leena Balakrishnan Sugumaran

**Affiliations:** 1 Pediatric Gastroenterology, Apollo Children’s Hospital, Chennai, IND; 2 Chemical Engineering, SSN College of Engineering, Chennai, IND; 3 Pediatrics, Apollo Hospitals, Chennai, IND

**Keywords:** breastmilk, early nutrition, feeding practices, formula feeding, infant feeding

## Abstract

Objective: This study aims to investigate infant and young child feeding practices in an outpatient setting in India.

Material and methods: About 103 parents of healthy children aged ≤6 years seeking outpatient pediatric care at an urban tertiary care hospital over one month were included in this observational study. Data regarding feeding practices was collected using a pre-designed and pretested questionnaire. Statistical analyses were performed using IBM SPSS Statistics for Windows, Version 26 (Released 2019; IBM Corp., Armonk, New York, USA) and Microsoft Excel (Microsoft Corporation, Redmond, USA).

Result: The average age of the study population was 21.26 ±16.561 months. About 45.6% of children were <12 months old. Around 42.7% of children were exclusively breastfed, 21.4% were formula-fed, and 35.9% were mixed-fed. About 49.15% of parents chose formula feeding voluntarily, and 50.85% due to inadequate milk supply. Around 71.2% were recommended by the clinician, 16.9% chose formula based on online information, and 11.9% chose autonomously. Nestle NanPro was the most used formula, followed by Similac Advance, Pediasure Advance, Danone, and NeoSure.* *Also,* *54.4% of parents monitored their child's growth. Nearly 15.5% and 6.8% of parents were concerned regarding insufficient weight and height gain, respectively. About 38.8% of parents introduced salt, sugar, or cow’s milk before their child reached one year.* *Around 62.1% of children were given vitamin supplements.

Conclusion: Our study revealed that most parents adhered to recommended guidelines by exclusively breastfeeding their children, which holds crucial significance in a developing country like India. Parents resorted to formula feeding only when necessary for optimal nutrition. Nestle NanPro was the preferred choice. While most parents demonstrated commendable awareness through growth monitoring and supplementation, there's a crucial need for campaigns to dispel misconceptions and promote proper feeding practices.

## Introduction

Nutritional adequacy is paramount for the optimal growth and development of infants. Human milk stands as the gold standard for infant nutrition, reinforced by the Indian Academy of Pediatrics, the American Academy of Pediatrics, and the World Health Organization [1]. In fact, understanding the composition of human milk and its benefits represents the most important advances in infant feeding research.

Infant formula is meticulously designed to serve as an efficacious substitute for breast milk, with a formulation aimed at emulating the nutritional composition of human milk. According to the Food Safety and Standards (Foods for Infant Nutrition) Regulations (2020), this standard encompasses infant formula in both liquid and powdered forms, intended for use, if necessary, as a substitute for human milk to meet the normal nutritional requirements of infants during the first six months. Such formulas are obligated to adhere to the stipulated requirements outlined in the infant milk substitutes, feeding bottles, and infant foods regulations [2]. Not surprisingly, the stringent quality and safety standards set for infant formulas surpass the majority of requirements established for other food products [3].

According to the National Health and Family Welfare (NFHS)-5 survey, only 63.7% of infants under the age of six months in India are breastfed [4]. Despite improvements in breastfeeding rates over the years, India has not met anticipated performance levels [5].

Employing appropriate supplemental milk mitigates the potential harm arising from insufficient milk. Expressing and storing breast milk serve as strategies to sustain breastfeeding when mother and infant are separated, contingent upon the preservation of its nutritional value. However, it is noteworthy that the total antioxidant capacity of expressed breast milk diminishes over time, particularly following refrigeration and freezing, as evidenced in the third-, seventh-, and 30th-day milk samples from Indian mothers [6]. Screened and pasteurized milk bank donor breast milk may present a viable option in certain cases, although the limited supply often restricts access to preterm and sick infants [7]. In some communities, animal milk may be the most readily available option, especially where long-standing traditions favor its use as supplemental infant feeding. However, its utilization requires fortifying additives and entails specific challenges ranging from the milking process to transportation, boiling, and dilution with boiled water and sugar in the case of cow's milk, along with the need for refrigeration or safe storage at home [8].

Considering availability, nutritional content, and bacteriological aspects, formula typically emerges as the optimal choice for supplemental milk. It is widely accessible and safe in areas where clean water and formula access are sustainable, barring dire poverty, endemic corruption, or extreme conditions that impede basic infrastructure development and disrupt essential supply access. Even under challenging circumstances, these risks can be mitigated through context-specific aid and education, such as guidance on obtaining appropriate water and ensuring hygienic preparation. Infant formulas are meticulously developed to maintain a balance of macro and microelements, offering more comprehensive nutrition compared to unfortified animal milk [9]. Therefore, while there is no universal solution applicable to every context, harm prevention is as straightforward as providing hungry infants with more milk, a solution that is widely feasible and adaptable to diverse circumstances [7].

In South India, limited research has been conducted on evaluating the dietary habits of young children. It is crucial for parents to be well-informed about appropriate feeding practices to enhance the health and immunity of their children. Regular assessments of infant and young child feeding practices within specific temporal and contextual parameters enable health professionals to comprehend the child's dietary habits and identify associated risk factors. This, in turn, allows for timely recommendations and modifications to be suggested to parents regarding their feeding practices. Consequently, we undertook a real-time survey to investigate the feeding practices in an outpatient setting in India.

## Materials and methods

This observational health study spanned one month (October 1, 2023-October 30, 2023) and involved 103 healthy children. The inclusion criteria were children aged ≤6 years seeking outpatient pediatric care at an urban tertiary care hospital in South India. Parents of children with co-morbidities such as congenital abnormalities, metabolic and endocrine disorders, and chronic conditions affecting the heart, kidneys, gastrointestinal tract, nervous system, blood, or any other medical ailment were excluded. Data collection employed a pre-designed and pretested questionnaire (Table 1), focusing on socio-demographic aspects (age and gender), feeding practices (exclusive breastfeeding, formula feeding, or mixed feeding), reasons for formula feeding, types of milk formula used, sources of information on formula, the introduction of salt, sugar, or cow's milk before the age of one, documentation of growth charts, parental concerns about the child's growth, vitamin supplementation, and vaccination schedules. Data entry and statistical analyses were performed using IBM SPSS Statistics for Windows, Version 26 (Released 2019; IBM Corp., Armonk, New York, USA) and Microsoft Excel (Microsoft Corporation, Redmond, USA), with results presented as percentages and numerical frequencies. This study was purely observational in nature, with no intervention, manipulation, or treatment administered to participants; thus, ethical clearance was not required for this type of research under prevailing guidelines.

**Table 1:** Survey questionnaire

Question	Your answer
Age	
Exclusively breastfed or formula-fed	Breastfed Formula fed Both
If formula fed indicated, why	Choice Short supply
Choice of formula	Self Online Recommended by pediatrician NA
What formula is the child taking right now	
Have you introduced salt, sugar, or cow milk under the age of one year	Yes No
Do you have a documented growth chart for your child	Yes No
Has there been any growth concerns regarding your child’s growth	Your answer
Child on any vitamins	Yes No Maybe
Vaccinations	Exclusive private Exclusive government

## Results

Data from 103 participants was analyzed.

Demographic characteristics

The average age of the study population was 21.26±16.561 months (mean±SD). Approximately half of the participants (45.6%; n=47) were below 12 months of age. Seventeen (16.5%) children fell within the 25-36 month age range, 16 (15.5%) were between 13 and 24 months, 14 (13.6%) were in the 37-48 month category, and nine (8.7%) children were aged above 48 months. Both genders were equally represented, with 52 (50.5%) females and 51 (49.5%) males included in the study (Table 2).

**Table 2:** Demographic characteristics of the study participants The data has been represented as n, %, and mean±SD

Age of child (mean±SD)=21.26±16.561 months		
Variable	Frequency (n=103)	Percentage
≤12 months	47	45.6
13-24 months	16	15.5
25-36 months	17	16.5
37-48 months	14	13.6
>48 months	9	8.7
Male	51	49.5
Female	52	50.5

Feeding practices

**Figure 1:**
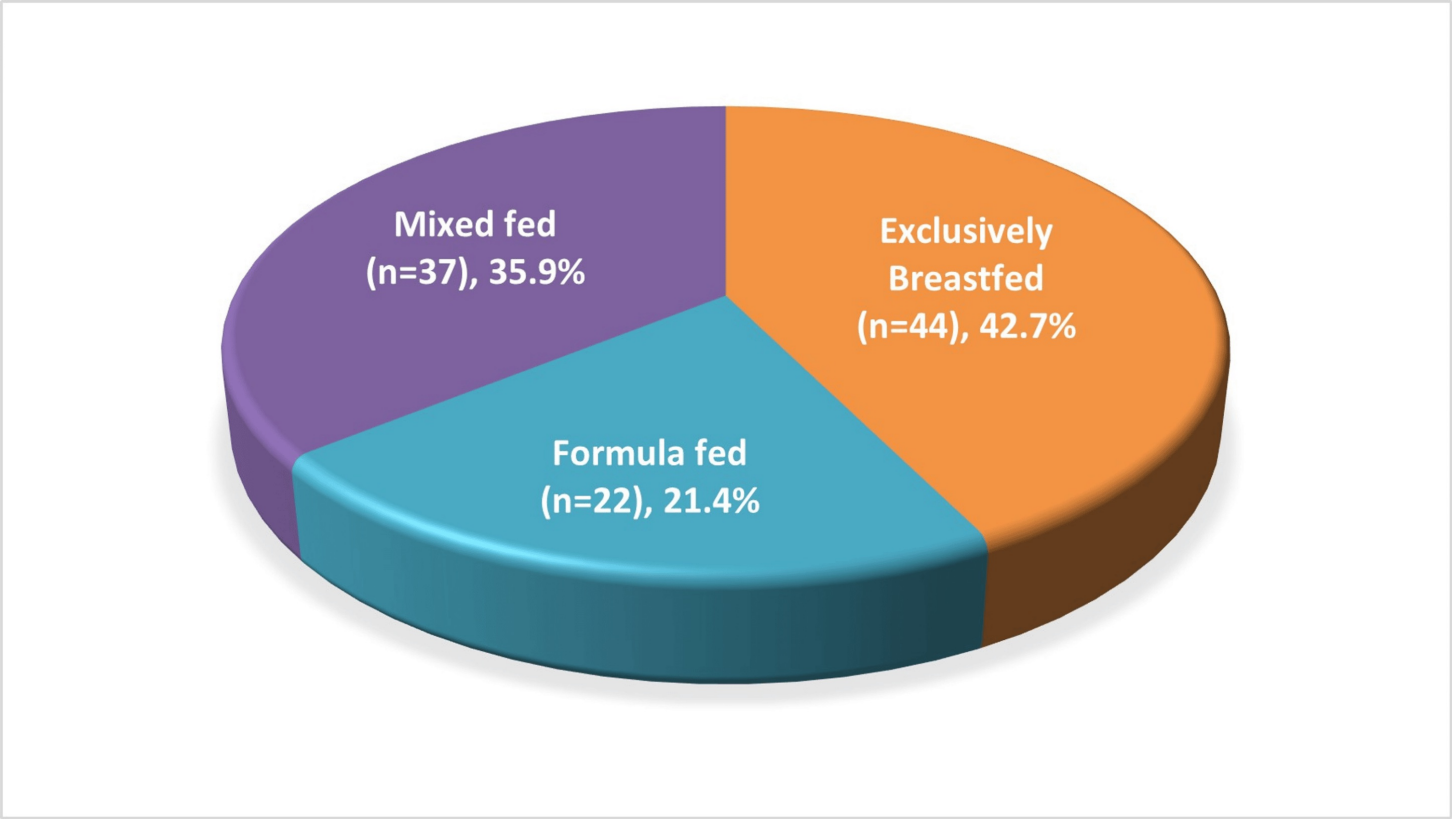
Feeding practices (percentage)

A predominant portion of the study population, accounting for 42.7% (n=44), was breastfed, while 21.4% (n=22) received formula feeding. Approximately one-third (35.9%; n=37) of the children were mixed-fed, involving both breastfeeding and formula feeding (Figure 1). The rationale behind choosing formula milk for formula-fed and mixed-fed children (n=59) was also assessed. Among the participants, 29 (49.15%) parents chose formula feeding voluntarily, and 30 (50.85%) mothers were compelled to resort to formula feeding due to inadequate milk supply. Additionally, four mothers (among the exclusively breastfed group) provided expressed breastmilk to their children. The decision for formula feeding was further probed, revealing that 71.2% (n=42) of the participants followed clinician recommendations, 10 (16.9%) made their decision based on online information, and seven (11.9%) parents autonomously made the choice (Table 3).

**Table 3:** Formula feeding practices The data has been represented as n and %.

Variable	Frequency (n=59)	Percentage
Reason for formula feeding		
Choice	29	49.15
Short supply	30	50.85
Choice of formula		
Self	7	11.9
Online reading	10	16.9
Clinician	42	71.2

**Figure 2:**
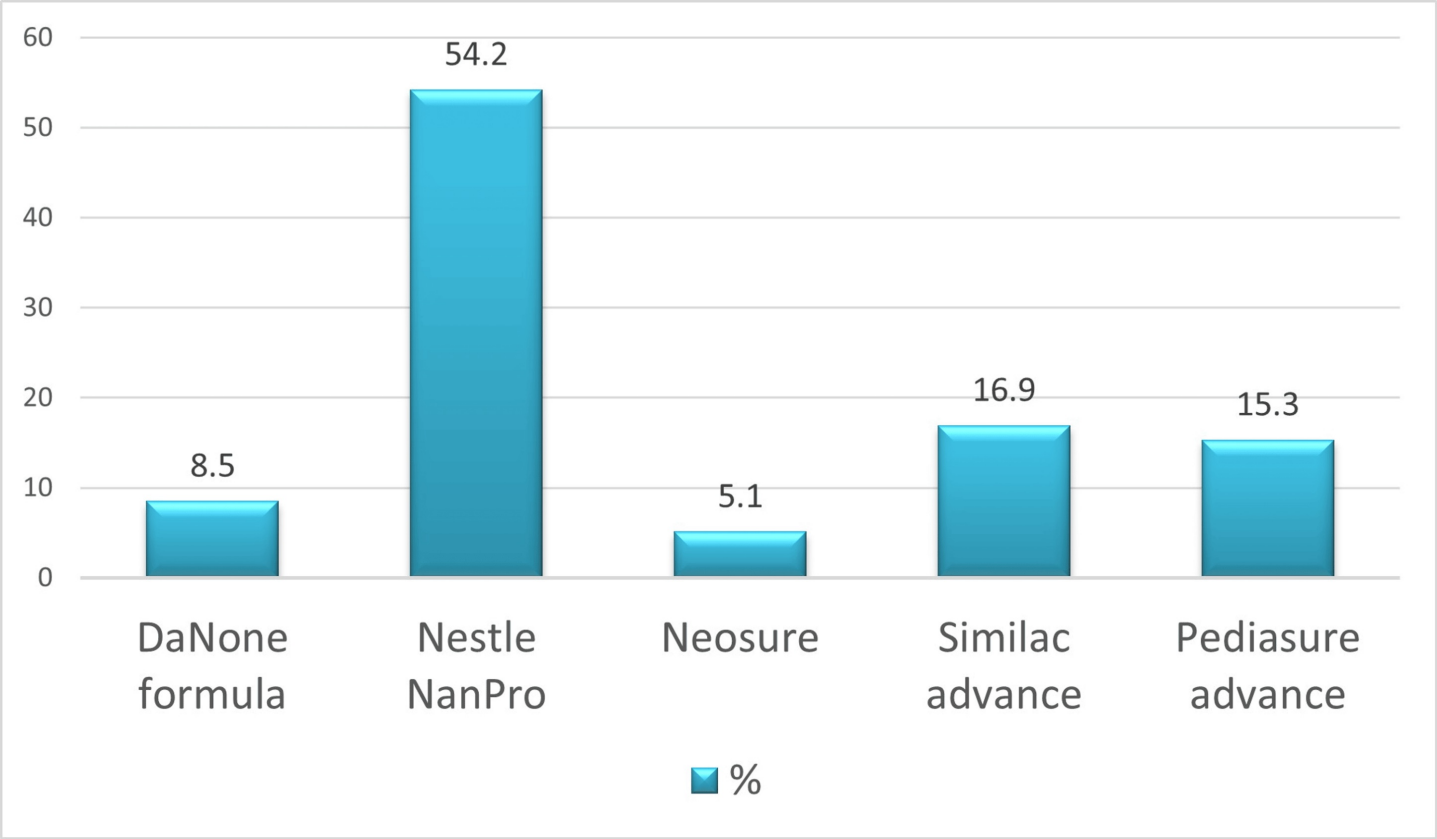
Commonly used infant formulas

In terms of formula brands, Nestle NanPro emerged as the most commonly utilized formula, chosen by 32 (54.2%) parents. Similac Advance was given to 10 (16.9%) children, Pediasure Advance was given to nine (15.3%), Danone formula was given to five (8.5%), and NeoSure was given to three (5.1%) (Figure 2).

Growth monitoring

The nutritional intake during infancy substantially influences growth. A majority of parents, constituting 54.4% (n=56), consistently monitored and documented their child's growth. Conversely, 45.6% (n=47) of parents did not regularly monitor their child's growth. Among the surveyed parents, 69.9% (n=72) expressed no apprehensions about their child's growth. However, concerns were noted among 16 (15.5%) parents regarding insufficient weight gain in their child, while seven (6.8%) parents were troubled by perceived inadequacies in their child's height. Additionally, eight (7.8%) parents conveyed apprehension about both the weight and height of their child (Table 4).

**Table 4:** Growth monitoring and concerns The data has been represented as n and %.

Variables	Frequency (n=103)	Percentage
Do you have a documented growth chart for your child?		
Yes	56	54.4
No	47	45.6
Have there been any growth concerns regarding your child's growth?		
Height and weight concern	8	7.8
Low weight gain	16	15.5
Poor height gain	7	6.8
None	72	69.9

Other feeding practices

We further investigated additional feeding practices prevalent in India. Notably, 40 (38.8%) parents introduced salt, sugar, or cow’s milk before their child reached 12 months of age, while 63 (61.2%) parents refrained from adopting this practice.

The majority of children, accounting for 62.1% (n=64) were administered vitamin supplements. Contrarily, 31 (30.1%) children did not receive any vitamin supplements, and eight (7.8%) parents were uncertain about the vitamin supplement status of their children.

In the concluding aspect of our examination, we assessed the vaccination status of the study population. Of the total, 60 (58.3%) children received vaccinations exclusively from private healthcare institutions, 20 (19.4%) obtained vaccinations solely from government organizations, and 23 (22.3%) received vaccinations from both private and government setups (Table 5).

**Table 5:** Other feeding practices The data has been represented as n and %.

Variable	Frequency (n=103)	Percentage
Have you introduced salt, sugar, or cow's milk under the age of one year		
Yes	40	38.8
No	63	61.2
Child on any vitamins		
Yes	64	62.1
No	31	30.1
Maybe	8	7.8
Vaccinations		
Exclusively private	60	58.3
Exclusively government	20	19.4
Both	23	22.3

## Discussion

Recognizing the critical importance of optimal feeding practices during infancy and early childhood, this timeframe is acknowledged as the "critical window" for promoting robust growth, overall health, and cognitive and behavioral development. In alignment with this understanding, we present an observational analysis of feeding practices within the relatively underexplored South Indian context.

Approximately half of the participants were below the age of 12 months, with an additional 15% falling within the 13 to 24 months range. Emphasizing the critical nature of feeding practices during the initial two years of life is imperative, as it serves as a foundational element for the development of cognitive, motor, and socio-emotional skills [10]. The predominant age group among the clinic attendees comprised children below the age of two years, aligning with the pivotal importance of feeding practices during the early stages of life.

The majority of children in the study exclusively received breast milk (42.7%), while nearly one-third (35.9%) were mixed-fed. A comprehensive analysis by Ogbo et al., based on the NFHS Survey, revealed substantial regional variations in the prevalence of exclusive breastfeeding across different regions of India. The prevalence of exclusive breastfeeding exhibited a decline with infant age, particularly notable in the South, where it dropped to 43.7% at five months [11]. Similarly, in a study by Karmee et al., 44.35% of infants were exclusively breastfed [12]. However, differing figures were reported in studies conducted by Patel et al. (55.9%) and Ganesan et al. (73.68%) [13,14]. The variation in exclusive breastfeeding rates among infants can be elucidated by diverse cultural practices, customs, and traditions prevailing in distinct geographical regions. Additionally, the retrospective design of the study may have introduced recall bias.

Approximately 40% of parents introduced salt, sugar, or cow's milk before their child reached 12 months of age. Genovesi et al., in their West Bengal-based study, noted a high percentage (91.5%) of infants having salt added to their foods at an early stage. Interestingly, infants who received added salt exhibited lower body weight measures standardized for height compared to those who did not. This association persisted even in 6- to 12-month-old infants fed kitchuri food [15]. Early salt intake during pediatric ages is linked to a high prevalence of arterial hypertension [16]. Similarly, added sugars in children below two years have been associated with cardiovascular disease risk [17], prompting recommendations from the European Society for Paediatric Gastroenterology, Hepatology, and Nutrition Committee to reduce free sugar intake, particularly emphasizing an upper limit of <5% energy intake in children ≥2 years and even lower in infants and toddlers <2 years. The recommended fluid for thirst in infants, post-introduction of solid foods, is water, and the avoidance of sugar-containing drinks in bottles or training cups is advised [18].

Rai reported that 26% of mothers in the study provided cow's milk as a substitute for breast milk [19]. A meta-analysis of 23 studies indicated that infants consuming cow's milk in the early years face a higher risk of iron-deficiency anemia compared to those consuming follow-on formula [20]. Prelacteal feeding has been associated with lower odds of receiving continued exclusive breastfeeding since birth [21,22]. The findings of this study can serve as informative material for advising mothers on the significance of delaying the introduction of salt, sugar, and cow’s milk in their infants' diet.

Despite the well-known benefits of breastfeeding, various external factors influence the decision, such as medical or physiological reasons like mammary hypoplasia, hormonal imbalances [23], or an infant diagnosed with galactosemia at birth [24]. Most mothers in our study opted for formula feeding due to a short supply of milk (50.85%). Other major reasons for discontinuing breastfeeding in the Indian subcontinent include issues with milk flow initiation, the mother becoming pregnant, prolonged periods of separation from the baby, breastfeeding-related pain, and maternal illness [14]. Nearly 4% of mothers in our study had offered expressed breastmilk to their children. Utilizing expressed breast milk stands as a healthful feeding alternative, particularly for postnatal mothers engaged in professional occupations. This pattern aligns closely with the 11% prevalence of expressed breast milk feeding observed in the Hyderabad study conducted by Rai [19].

In situations where breastfeeding is not feasible or when milk is unavailable, formula feeding serves as the primary dietary source [24]. In our study, 49.15% of parents consciously opted for formula feeding. A significant proportion (71.2%) selected formula based on recommendations from healthcare professionals, approximately 16.9% relied on information obtained through online sources, and around 11.9% independently made the decision. Among formula options, Nestle NanPro emerged as the most favored, followed by Similac Advance, PediaSure Advance, Danone formula, and NeoSure. Over the past 50 years, significant progress has been made in the development of infant formulas, marked by numerous clinical trials. Synthetic infant formula, to a certain extent, replicates the nutrient composition of human milk. Lactoferrin, osteopontin, lutein, human milk oligosaccharides (HMOs), milk fat globule membrane (MFGM), and docosahexaenoic acid (DHA) have been incorporated into commercial infant formulas based on published evidence. Research on these nutritive and non-nutritive components highlights their beneficial effects on gastrointestinal health and neurodevelopment during early infancy [24].

Most parents, exceeding 50%, actively monitored and documented their child's growth. Concerns regarding inadequate weight gain were reported by 15.5% of parents, while 6.8% expressed concerns about insufficient height gain. Monitoring a child's growth is crucial for identifying deviations from the normal growth trajectory [25]. Incorporating growth monitoring into routine pediatric care, with regular intervals throughout childhood, plays a vital role in detecting nutritional deficiencies, endocrine disorders, and chronic systemic illnesses at an early stage [26]. The process is quick, easy, inexpensive, and noninvasive, offering valuable insights into the overall health of children [25]. Whether through breastfeeding or formula feeding, both approaches supply sufficient nutrients to support normal growth and development up to 72 months [27].

More than 60% of the children in our study received vitamin supplements. This contrasts with the findings of Ganesan et al., where only 30% of children were administered supplements; it's noteworthy that the study population in their research was confined to those aged 12-24 months [14]. According to NFHS-5, a significant 67.1% of children aged 6-59 months exhibit anemia (hemoglobin level <11.0 g/dl) [4]. Vitamin D and vitamin A deficiencies are also prevalent among Indian children. In public health programs, the provision of micronutrient supplements is considered a short-term strategy to complement dietary approaches aimed at alleviating micronutrient deficiencies [28,29]. Embracing a substantial rate of vitamin supplementation could serve as a favorable strategy to prevent the detrimental effects of micronutrient deficiencies.

Strengths of the study

The study's strengths lie in the utilization of validated questionnaires by the investigators and the real-world inclusion of all healthy subjects, mitigating the potential for selection bias.

Limitations of the study

Given the retrospective nature of the study, reliance on recall methods introduces the possibility of overestimation or underestimation in measuring the child's feeding practices, stemming from recall and social desirability biases.

## Conclusions

Infant formula consumption has witnessed a substantial surge in the contemporary era, necessitating the development of formulas tailored to meet the growth and developmental needs of infants unable to breastfeed. Our observational analysis revealed that Nestle NanPro emerged as the favored formula among parents in the study. Most parents diligently monitored their child's growth and administered vitamin supplements, underscoring a commendable level of awareness among caregivers. However, there is a critical need for public awareness campaigns to counteract misconceptions regarding the use of salt, sugar, and cow’s milk in early life and disseminate accurate information regarding appropriate infant feeding practices. Interventions in health systems should explicitly target caregiving practices, emphasizing the imperative role of care and feeding. Improvements in growth monitoring should be prioritized.
